# Adult social care nursing: A hybrid concept analysis

**DOI:** 10.1016/j.ijnsa.2026.100491

**Published:** 2026-01-24

**Authors:** Claire Pryor, Siobhán Kelly, Vanessa Heaslip, Deepa Korea, Melanie Stephens

**Affiliations:** a School of Health and Society, University of Salford, Mary Seacole Building, Salford, Greater Manchester M6 6PU, UK; b RCN Foundation, UK

**Keywords:** Social care, Nursing, Concept analysis, Health and social care, Registration

## Abstract

**Objective:**

The concept of adult social care nursing is poorly articulated in the literature. This study explores and defines adult social care nursing by examining its antecedents, attributes, and consequences. By clarifying this concept, the research aims to enhance understanding among policymakers, educators, service providers, and the public, fostering greater recognition of social care nurses' roles, workplaces, and contributions.

**Design:**

A qualitative hybrid concept analysis model was used. This approach combined theoretical examination with empirical inquiry to develop a comprehensive understanding of the concept. The study was structured in four phases: concept identification, literature review, empirical data collection, and integrative analysis.

**Data sources:**

A comprehensive literature search was conducted using CINHAL, Medline, APA PsycInfo, Wiley Online, OVID, and The King’s Fund library. Grey literature was explored through the Department of Health and Social Care’s adult social care collection, expert consultation, and reference handpicking.

**Participants:**

Nineteen participants took part via four focus groups, two 2:1 interviews, and one 1:1 interview. Eighteen participants were registered nurses, and one participant was a registered social worker with experience of working with nurses.

**Methods:**

Using a hybrid concept analysis approach, the study progressed through multiple phases. A preliminary exploration (Phase 1) provided an initial conceptual framework, which was refined through a literature review (Phase 2). Empirical fieldwork (Phase 3) involved focus groups and interviews, thematic content analysis was utilised to identify key attributes, followed by integrative analysis to synthesise findings and refine the conceptual model. The study was not registered in a trial registry given it is a qualitative study.

**Results:**

This study identified that adult social care nursing is present when people have a combination of health and social care needs that require registered nurses’ care. Attributes include a career of choice, independent and autonomous nursing, with professional and business skill development including advanced practice. This supports social care nurses in their goal to be dynamic change agents who empower people to be active participants in their own care, with improved quality of life, being able to live well with their health needs in a social context.

**Conclusion:**

This paper proposes an initial definition of nursing within adult social care, which serves as a foundation for further discussion and development. It highlights nurses' vital role, diverse skill set, and equal partnership in the social care landscape, reinforcing their significant contributions to integrated, person-centred care across diverse settings.

**Social media abstract:**

Defining adult social care nursing: A dynamic field where registered nurses manage complex needs, and drive person-centred, evidence-based care across diverse settings.

**What is already known**

• Adult social care is a well-established concept, but nursing within this sector lacks definition, leading to fragmented understanding of nurses’ contributions and responsibilities.
• Nurses working in adult social care often feel unseen, with their skills perceived as having less value compared to nurses in other healthcare settings.
• A clear understanding of nurses' roles in adult social care is crucial for strengthening health and social care integration and ensuring their contributions are fully recognised.

Alt-text: Unlabelled box dummy alt text

**What this paper adds**

• The first formal definition of adult social care nursing, serving as a foundation for further discussion and development.
• Highlights the complexity, autonomy, and expertise of social care nurses, challenging misconceptions and emphasising their role as equal partners in integrated health and social care.

Adult Social Care Nursing: A Hybrid Concept Analysis

Alt-text: Unlabelled box dummy alt text

## Introduction

Despite adult nurses being a distinct occupational category of health workers delivering care in a variety of institutions and the home (Abbott and Wallace, 2020), little is known about the 40,000 registered nurses who work in the adult social care sector in the United Kingdom (UK) (Cornes and Manthorpe, 2022). The term ‘social care’ is widely recognised, having been used for over 20 years as an evolution of UK social service provision. Its origins lie in the integration of ‘social services’ and ‘community care’ (Needham et al., 2024). Initially, adult social care was categorised as care provided to people with varying needs due to disability, frailty or older age (Needham et al., 2024), but more recently extended to include needs due to physical and/or mental illness or impairment (Longo et al., 2023). This usually encompasses personal care and may not require a registered nurse; is not for health-based needs and is provided by carers or personal assistants. Internationally, this provision is referred to as ‘long term care’ (National Institute on Aging, 2023), however the term ‘social care’ is becoming more commonplace, for example in Australia, Canada and Ireland (Moscovitch and Thomas, 2018; Health Service Executive, 2024; Needham et al., 2024).

It is important to acknowledge that ‘adult social care nurse’ and ‘adult social care nursing’ are relatively new terms, predominantly used in England, and may not be recognised universally. This paper uses them to discuss nurses, and nursing practice which happens outside of the UK National Health Service employment contract structure. Adult social care nursing sits in a unique position, with nurses as registered healthcare professionals, engaged in nursing care whilst being embedded within social care services and philosophies. In this paper we use the term *adult social care nurse* to refer to the practitioner, and *adult social care nursing* to describe the associated practice. This approach aligns with nursing nomenclature conventions while drawing attention to the interplay between practice, environment, and the professional registration that underpins this work.

The UK has a well-established policy and governance environment pertaining to adult social care, as well as an integrated health and social care system (Uribe et al., 2023), positioning it as a global leader in this field. Current adult social care provision acknowledges the complexity of care, which may encompass both short-term and long-term support throughout the life course. This may take place in a variety of settings such as person’s home, day centres, care homes, and reablement services (The King's Fund, 2025). It can also include care for people with multimorbidity and vulnerable populations (Uribe et al., 2023), such as people who are experiencing homelessness or domestic violence (Daly, 2020).

Following the Covid-19 pandemic in the UK, there has been a significant drive to integrate health and social care systems (Department of Health and Social Care, 2022a). These efforts have faced challenges around equitable integration, particularly ensuring that the ‘social’ element is given equal consideration alongside integrated (community and hospital) health provision (Uribe et al., 2023). With the ongoing 10-year plan for Health in England (Department of Health and Social Care, 2022b) focusing predominantly on the NHS goals there is a growing concern that equal attention should be given to the social care system in which some of these changes will be implemented. It is increasingly clear that the success of the health plan depends on greater integration between health and social care systems (The King's Fund, 2024), including nursing. Positive steps are being taken, for example, the establishment of an independent commission to build cross-party consensus for a National Care Service, however while these are promising developments, it remains unclear where adult social care nursing fits and how the role, its needs, and professional requirements will be addressed.

Part of this complexity stems from the limited understanding of the role of registered nurses as healthcare professionals within social care settings. While the concept of adult social care is well-established, the concept of nursing within this context remains underdeveloped. There is a need to explore and articulate the types of care registered nurses provide in social care settings to enhance clarity and recognition of their contributions.

Within the UK, nursing is regulated by the Nursing and Midwifery Council, who set standards of proficiency for registrants. They state that nurses must:

…Be able to care for people in their own home, in the community or hospital or in any health care settings where their needs are supported and managed (Nursing and Midwifery Council, 2018)

It is here that we find the nexus between registered nurses as health professionals, in social care employment. There is a convention to identify nurses in terms of role or location of care delivery (such as ‘cardiology nurse’). This nomenclature has expanded to include ‘social care nurse’ more recently. However, there is lack of clarity around what is meant by ‘social care nursing’, who ‘social care nurses’ are, what they do, and where they work. Even though they may be delivering care based on a health need, which has been assessed, and remains funded through National Health Service and integrated commissioning services, the lack of clarity pertaining to the work of adult social care nurses may negatively influence UK health and social care policy, education, and services.

This study aims to explore and define the concept of adult social care nursing by examining its antecedents (prerequisites), attributes (characteristics) and consequences (outcomes) (Walker and Avant, 2011). It presents a ‘state of the art’ of adult social care nursing, at a particular point of time, rather than offering a definitive, static answer (Rodgers, 2020). By clarifying the meaning of “adult social care nursing”, this research seeks to inform policy, commissioning, education, and service delivery by making the role, scope, and contribution of nursing in adult social care more visible and better understood.

### Study design

Concepts are “complex mental formulations of experience” (Chinn and Kramer, 1991, p. 58) and centre around a particular topic or practice. This study aims to unpick and expose the elements that constitute adult social care nursing. The study was conducted using the qualitative hybrid concept analysis model (Schwartz-Barcott and Kim, 2000), which combines theoretical analysis with empirical data, supporting robust identification of concepts grounded in both literature and lived experience. Recognising the interplay between these elements, a hybrid approach provides a robust framework, drawing on qualitative data from those with direct experience of adult social care nursing. This helps confirm or refine assumptions derived from analysing the literature and deepen overall understanding.

**Fig. 1:**
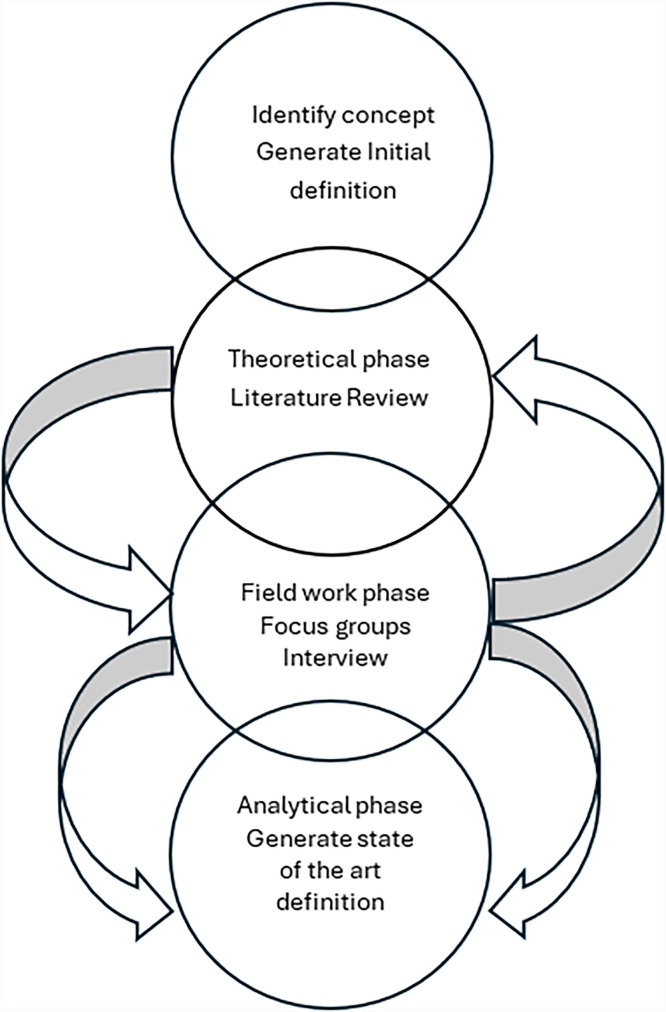
The hybrid analysis process.

The study design consisted of four overlapping phases presented in Fig. 1 and below:

1. Phase One Initial Concept Identification – The researchers began by identifying the concept of adult social care nursing based on their existing knowledge and drafted an initial definition.
2. Phase Two Literature Review and Analysis – A review of available literature was conducted to explore existing definitions and examine how the concept is described. This phase also involved the early identification of composite antecedents.
3. Phase Three Fieldwork with Participants – Engagement with participants was undertaken to validate, refine, or challenge the evolving understanding of the concept.
4. Phase Four Final Integrative Analysis – Data from all phases were synthesised to develop a comprehensive definition, detailing the concept’s antecedents, consequences, and attributes.

Rather than adhering to the traditional hybrid analysis approach, the study used focus groups instead of participant observations. This methodological choice aimed to foster in-depth discussions from the perspective of nurses, allowing for a richer exploration of the concept.

The study team consisted of registered adult nurses with research, education and clinical experience in social care, community nursing and inclusion health settings (1, 2, 3) and a sociologist with expertise in care home and third sector research (4). All team members were experienced qualitative researchers. Reflexive practice was employed throughout to critically consider how researchers’ assumptions, experiences, and positionalities may have influenced the research process, interpretation, and transferability of findings.

## Ethics

The study received ethical approval from the University of Salford ethics committee (Study Reference 1674). Participants in the fieldwork phase were provided with detailed information sheets and had the opportunity to discuss the study with the research team to ensure informed consent. They signed consent forms before any research activities began.

## Methods

### Phase one initial concept identification

Adult social care nursing was identified as the concept for investigation. The term adult social care nurse has increasingly been used in the UK to describe registered nurses working outside the National Health Service, including those employed in long-term care facilities, home care services, and charitable organisations providing nursing care within a social care context. This growing recognition of nursing roles within adult social care has been supported by national policy developments, notably the establishment of the English Social Care Nurse Advisory Councils, led by the Chief Nurse for Social Care.

Despite the increased use of the term social care nurse, the research team identified persistent difficulty in articulating what adult social care nursing actually entails. Informal appraisal of relevant literature and policy documents revealed a lack of a clear or consistent definition of the role. This absence of conceptual clarity highlighted the need for a focused examination of adult social care nursing as a distinct concept. The practice-oriented and experiential origins of this inquiry align with Schwartz-Barcott & Kim’s (2000) recognition of the value of concepts that emerge from practice-based encounters, particularly those characterised by complexity and the emotional challenges associated with explaining professional roles to others.

As part of the hybrid analysis process, the study began with (1) developing a tentative summary definition of adult social care nursing based on the teams professional nursing knowledge and experience of working with, and alongside, a variety of social care nurses. This served as a foundation for guiding and refining the literature review process and is presented below.

• *Adult social care nursing happens in care homes and some wider settings. This may include some specific National Health Service funded community services, but their involvement is unclear.*
• *Adult social care nurses care for people who are predominantly older and living in care homes, with health needs that require care from a Nursing and Midwifery Council registered nurse.*

### Phase two literature review and analysis

The literature search adopted an exploratory scoping approach, reflecting the unrefined nature of the construct under investigation and the dynamic policy context within the UK. The search was designed to capture a broad range of evidence, including empirical studies, policy documents, reports, and grey literature. Given the heterogeneity of the material reviewed, all sources were collectively referred to as “records” to encompass the full range of literature included.

Searching commenced prior to, and continued throughout the qualitative focus groups and interviews building the definition in keeping with Hybrid concept analysis theory (Schwartz-Barcott and Kim, 2000)

The search string 'adult social care' AND ‘nurs*’ was applied across multiple electronic databases and collections, including CINAHL, Medline, APA PsycInfo, Wiley Online Library, OVID, the King's Fund Library, and the UK Department of Health and Social Care adult social care collection. In congruence with the exploratory nature of this review, the aim was to understand the breadth and diversity of available evidence (as opposed to limiting the review through a defined set of indexed terms). Therefore, natural language/free text search terms were used, recognising that reliance on MeSH terms may limit retrieval of relevant sources in an exploratory review, as MeSH terms may not be available and well developed for new and emerging topics and concepts of interest (Leblanc et al., 2024).

A ten-year time limit was set to ensure the literature reflected contemporary practice, policy changes, and governance.

### The inclusion criteria were

• Publications in English
• A UK focus or context
• Full-text availability
• Publication date range: 2014–2024

To ensure a comprehensive understanding of how the term has been used, no records were excluded based on type of publication (e.g. editorial or commentary) or subjected to methodological quality ratings. However, records were excluded if they did not explicitly describe registered nursing activities or settings. Records that referenced broader literature but contained a clearly distinguishable UK component were screened and considered for inclusion, provided they included relevant discussion and commentary. To capture grey literature, a combination of reference tracking, targeted grey literature searches, and expert consultation was employed, ensuring a more comprehensive exploration of the topic.

### Analysis

**Fig. 2:**
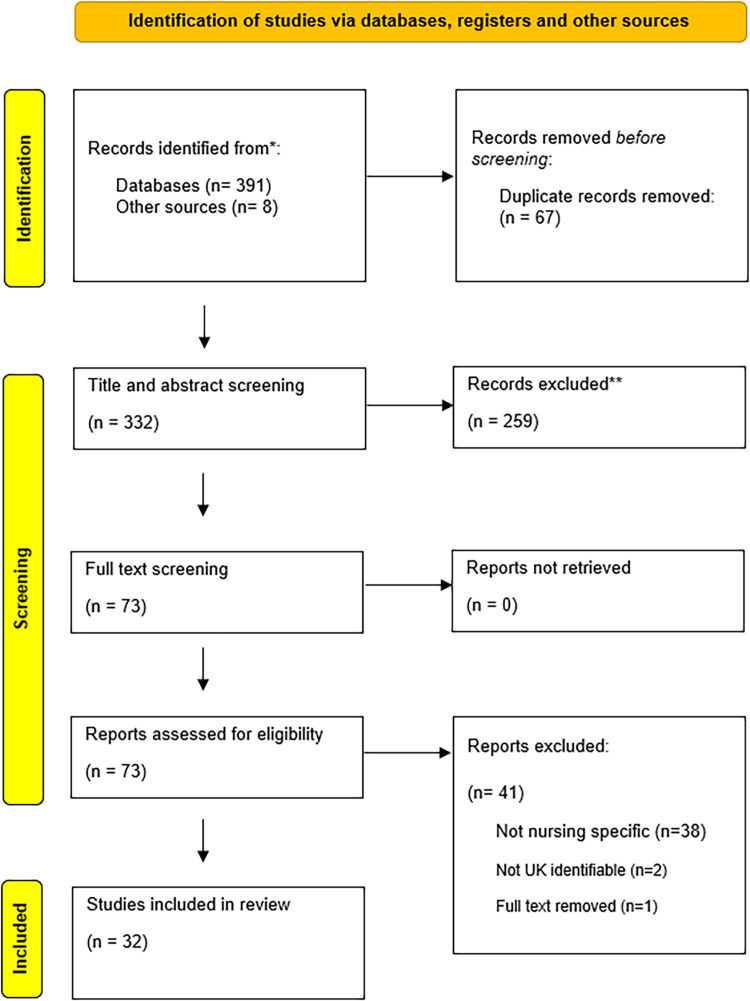
Paper selection process (PRISMA 2021 flow diagram (Page et al., 2021)).

The literature search was undertaken between October and November 2024. One researcher (1) completed the initial search and screening of record titles, abstract, (executive summary, preface; or for commentary papers an initial scan read). A 10 % proportion of records screened by 1 were assessed by a second reviewer (2). Full text screening was completed by 1 and 10 % of records classed as include, exclude and borderline were reviewed by (2) and discussed with (1) to reach consensus. Individual records which stemmed from the same data set were assessed by two members of the research team (1 and 4) in terms of additional information or areas covered and consensus reached around inclusion. Thirty-two records were selected for analysis. Fig. 2 details the selection process.

A total of 32 records were included in the review. Records fell into three broad groups: (1) research studies, including empirical studies and literature reviews (*n* = 15); (2) standards for education (*n* = 1), commentary pieces, or information guides written for a nursing audience (*n* = 12); and (3) sector reports (*n* = 4).

Research records comprised qualitative studies (*n* = 5) and review papers, including scoping reviews (*n* = 2), a rapid evidence review (*n* = 1), a realist synthesis (*n* = 1), and an undetermined literature review (*n* = 1). Mixed-methods studies (*n* = 4) and one quantitative study were also identified.

Each record was then read by author (1), who appraised for both overt and subtle accounts of core components, including identifiable antecedents (factors that must be in place before a concept can occur), attributes (characteristics of a concept), and consequences (what happens because of a concept) (Walker and Avant, 2011; Hellmand, 2024). Summary notes of emerging empirical referents (Walker and Avant, 2011), considerations linked to antecedents, location or context of nursing activity, attributes and consequences were systematically extracted by author (1) and recorded in a Microsoft Excel spreadsheet next to direct quotes and key text. Complexities with the reports were noted (such as political or contextual shifts) and links to any associated concepts or provision (such as district or community National Health Service nursing). Researcher notes were kept alongside each records’ assessment to support further analysis during the fieldwork phase and final integrative analysis. An example is provided in Supplementary File 1.

**Fig. 3:**
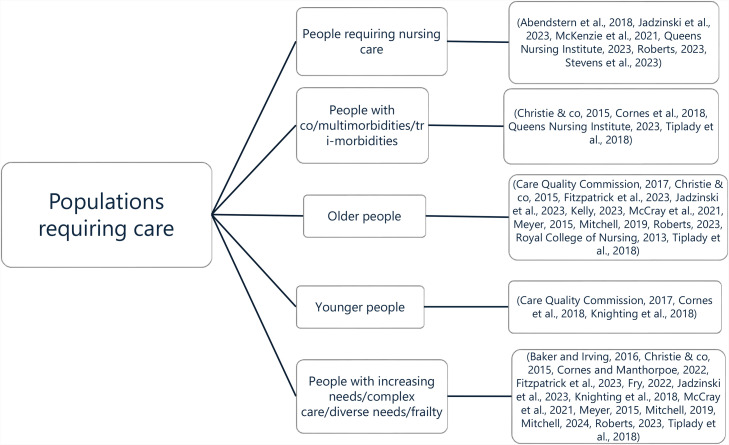
An initial antecedent grouping for “populations requiring care”. (Abendstern et al., 2018; Baker and Irving, 2016; Christie & Co, 2015; Fitzpatrick et al., 2023; Fry, 2022; Jadzinski et al., 2023; Kelly et al., 2023; Knighting et al., 2018; McCray et al., 2021; Meyer, 2015; Mitchell, 2019; Mitchell, 2024; Roberts, 2023; Stevens et al., 2023; Thompson et al., 2016).

After each record had been appraised, the next step of the analysis included examining the clustering of attributes and contextual variations. Labels were applied to the data to create an audit trail of groupings. These groupings were then reviewed, merged, and reorganised to establish the key elements of the concept. Author (2) independently reviewed these groupings. Differences in interpretation were discussed until agreement was reached. This triangulation served as our consensus procedure for confirming and refining the categories. Emerging factors were charted and grouped while preserving the original terminology used to ensure transparency in concept development (Fig. 3 provides an example of an initial antecedent grouping).

### Phase three fieldwork

To corroborate, expand, or refine the initial understanding identified from the literature (Schwartz-Barcott and Kim, 2000), the fieldwork phase collected descriptions and commentary from individuals who self-identified as working in adult social care nursing settings or roles. This phase overlapped with the literature review, ensuring a comprehensive, dynamic approach to understanding the concept.

We did not attempt to define what constitutes an 'adult social care nursing' role or setting. Participants were recruited through purposeful and snowball sampling, primarily via online social media adverts targeting a broad range of individuals who consider themselves to work in, with, or for social care nursing services. Additionally, direct emails were sent to Social Care Nursing Advisory Councils and nurse leaders with a strategic overview of nursing services in social care. Information was also widely shared through professional networks.

## Participants

Potential participants were asked to email (1) to arrange further discussion or participation in a focus group or interview based on their preference and availability. This facilitated maximum participation. Follow-up emails and adverts were posted at timed intervals and upon requests for further information or recruitment needs. Recruitment continued through data collection and analysis until no new themes were evident in the nurses’ discussions. This was in keeping with the principles of data saturation; however, the authors acknowledge that the decision to stop data collection is “inescapably situated and subjective” (Braun and Clarke, 2019), linked to the researchers’ own perceptions and experiences (Bowen, 2008).

19 participants were recruited: Eighteen participants were registered nurses, and one participant was a registered social worker with experience of working with nurses. Participants were recruited from across the UK and included nurses working in single, multi-site, and regional roles and locations. Nurses reported a variety of employment foci, spanning direct care provision, deputy manager and manager responsibility, regional operational roles, and some working with national regulatory bodies or oversight groups. There was representation of both UK and internationally educated nurses, holding a range of educational qualifications, and included all Nursing and Midwifery Council fields of practice (adult/mental health/learning disability/children). Several participants were dual registered or had been on several parts of the Nursing and Midwifery Council register. All the nurses held at least one Nursing and Midwifery Council registration at the point of data collection.

All participants provided signed consent forms, and verbal consent was reaffirmed at the start of interviews/focus groups.

### Data collection

Data was collected by authors (1) and (4) across four focus groups, two 2:1 interviews, and one 1:1 interview which was attended by author (1) alone. Focus groups averaged 56 min and were hosted, recorded, and transcribed via Microsoft Teams.

Questions were purposefully designed to elicit discussion which would show elements of attributes, antecedents, and consequences. Participants were asked to introduce themselves, and describe their links or role in social care nursing, and they were then asked opening questions designed to explore:

• The ‘theme’ or ‘what’ of social care nursing- what it is or is not
• ‘How’ nursing is undertaken in this area
• What the phrase ‘adult social care nursing’ meant to them
• Who they thought was cared for in adult social care nursing settings
• Whether it is different or the same to other forms of nursing (e.g. nurses in the National Health Service)

Notes were taken throughout by (1) and (4), with (1) leading discussions. The focus groups were situated as a conversation between the participants and researcher to glean specific information (Polit and Beck, 2014) whilst summarising sections and key points back to participants. This supported clarification of understanding, checking that the summaries represented participants’ thoughts and opinions, and probing further to support additional elaboration or refinement (Rubin and Rubin, 2012). This process of member checking in action supported a shared understanding and validity of interpretations made throughout the discussion (Moule et al., 2017). Throughout, careful attention was paid to ensure all voices were heard, and to manage any instances of dominant personalities (Finch et al., 2014), with (1) ensuring all participants were supported to actively contribute. Transcriptions were cleaned and checked for accuracy by (1), who rewatched and listened to all recordings following the sessions. All recordings were transferred from Microsoft Teams to a secure research team server, and participants were assigned unique identifier codes for use throughout the process.

## Data analysis

One researcher (1) conducted analysis of the focus groups and interviews, with (4) reviewing key areas for discussion, clarification and interpretation. Analysis began after the first focus group and proceeded concurrently. An inductive thematic analysis approach was used, given the lack of preexisting empirical work exploring the nature of adult social care nursing (Hsieh and Shannon, 2005; Braun and Clarke, 2006). (1) read and reread the data, noting key messages, summaries, and recurring themes.

Notes taken at the time of interview by authors (1) and (4) were reviewed alongside the transcripts, and any nuanced interpretations or contextual meanings were integrated into the transcript data. Individual charting tables were developed by author (1) for antecedents, attributes, and consequences. These tables were populated with participants’ key information and illustrative quotations, the context in which issues were discussed, initial interpretations or codes, and subsequent refinements.

Once populated, the charting tables were reviewed by author (1) in relation to the study’s overarching objectives and organised into antecedents, attributes, and consequences. Following appraisal of all transcripts, a process of summary refinement was undertaken, resulting in the development of thematic statements across the dataset. An example of this analytic process is provided in Supplementary File 2.

### Phase four final integrative analysis

In the final phase of the study, the findings from both the theoretical and fieldwork phases were drawn together by author (1), who read them in parallel, to form a cohesive account of the concept of adult social care nursing. Summaries were drafted, re drafted, condensed, and reviewed by all members of the research team. The final summary was again reviewed against the findings of the literature and fieldwork phases to ensure full integration and congruence of the data sets. This process led to the consideration of, and development of professional “state of the art” definition of adult social care nursing, encompassing its antecedents, attributes, and consequences, and a short public facing summary definition.

### Findings from the literature review

The literature review highlighted that there were no published definitions of adult social care *nursing*, and there was an absence of clarity around what social care was, and its empirical referents. Some papers offered partial, incomplete or brief opening summaries, or offered narratives around social care more broadly (Royal College of Nursing, 2013; Care Quality Commission, 2017; McKenzie et al., 2021). In addition, the Queens Nursing Institute (Queens Nursing Institute, 2022) contextualised adult social care nursing as happening in care homes or local authorities and provides education standards for specialist practice. However, narratives around the full scope and working environments of nurses in social care appear lacking.

### Antecedents

Antecedents for adult social care nursing centred on groups of people, or individuals who have a combination of health and social care needs that required registered nurses to support. The predominant recipient of nursing care was identified as older people. However, some literature did include younger people requiring nursing care. Factors influencing nursing care requirements included people living with learning disabilities, mental health needs, physical disabilities and often co, multi, or tri-morbidity.^1^ Care locations and the duration of nursing care provision also form key antecedents. Care was identified as being delivered across longer-term settings (such as care homes), shorter-term, or intermediate care settings. Nursing roles were primarily found in care home settings and domiciliary care. They also extend into wider environments, including intermediate care services for people experiencing homelessness, memory assessment clinics, palliative care settings such as hospices, and more broadly within local authorities.

### Attributes

The attributes of nursing in adult social care centre on the ethos of nursing practice, the complexity of roles, and the application of business skills. A key focus is the provision and promotion of individualised care, guided by a person-centred or relationship-focused philosophy. Another significant attribute is the promotion of health, social well-being, and independence, along with providing essential support to carers and family members. This requires collaboration across organisations, agencies, and professional groups, fostering integrated and coordinated care. The literature highlights that nurses in adult social care often undertake complex roles, requiring a high level of professional autonomy and expertise including specialist or advanced nursing skills. These skills enable them to manage and monitor conditions effectively and respond appropriately. Additionally, many nurses in adult social care take on managerial responsibilities that demand business acumen, advanced decision-making skills, and regulatory oversight. Adult social care nurses described in the literature display attributes that support personhood and dignity, underpinned by advocacy and the ability to facilitate shared decision-making. They are also educators, supporting staff development and contributing to the wider professional community.

## Consequences

The consequences of adult social care nursing are presented for both the individuals receiving care and the nurses themselves. For people using services, the outcomes include high-quality care that supports, maintains, or enhances their quality of life. Individuals are actively included as partners in care decisions, goal setting, and the promotion of autonomy, with care provision guided by person-centred, person-led, or holistic principles. Nursing care is delivered by those who know the service users well. This familiarity fosters personalised care and strengthens relationships, contributing to better outcomes. Additionally, carers (e.g. family members) feel supported within these settings and benefit from the collaborative and holistic approach taken by the nurses.

For the nurses working in adult social care, the consequences include opportunities for career progression, the development and delivery of advanced nursing practice, and the ability to shape the future workforce through student placements and education. Nurses in adult social care settings also could act as innovators and change agents through participation in research, evidence-based practice, and policy. More broadly, adult social care nursing plays a crucial role in strengthening the interconnectivity of services, often requiring collaboration across complex organisational and professional boundaries.

### Findings from the fieldwork

Below we present the antecedents, attributes and consequences of adult social care nursing as highlighted by the participants. Before presenting these findings, it is important to first acknowledge that some participants questioned the nomenclature of nursing, typically undertaken by identifying nurses by location of service or focus of nursing role (e.g. adult social care nurse/cardiology nurse). Some questioned the need to ‘environmentalise’ or categorise nursing in such a way, highlighting that all registered nurses hold NMC registration, and as such do not see the need to name themselves past ‘registered nurse’.

*I think it's just nursing, put it sort of simply like that. It's just a different setting…(P16)*

Whilst this formed a valuable part of the discussion, it was not universal. For other participants the naming of their work environment seemed to instil pride, clarity, and supported an identification of their skill and expertise, calling for it to be a recognised field of nursing practice.

*I think there should be a speciality for social care nursing and that will be the speciality of specialities (P11)*

### Antecedents

The antecedents of adult social care nursing were identified as the presence of individuals requiring nursing care throughout the life course, necessitating the involvement of Nursing and Midwifery Council registered nurses across a diverse range of settings.

*So, we have nurses who work as nurses within our home, which is a neurological nursing home specifically around Huntington's [disease], acquired brain injury, etcetera. We have nurses who work in our mental health division, who again support people within supported living flat based schemes, …(P7)*

Participants described this care as occurring wherever people reside, situated within complex health and social care systems. These systems often span multiple regulators, service models, and infrastructures, requiring coordination and adaptability to meet the needs of individuals in various environments.

*we support people in their own homes or in temporary accommodation arranged by social services.... The person may be looked after in an Airbnb, in a caravan… (P19)*

*The funding for complex care comes from the NHS and the social care, so you have a bit of …both… in complex care at home we've got a foot in both camps, and it doesn't always fit nicely in into a box… (P14)*

### Attributes

Four attributes were evident: career of choice, independence and autonomy, a personalised ethos to care provision, and a fusion of nursing and business skills.

### Career of choice

The participants reported that their decision to work in social care nursing was a conscious choice. Many entered this field after gaining extensive post-registration experience, while others chose it directly upon qualification. This decision was active, aligned with their professional values, and driven by a desire to apply their skills in a setting where they felt they could make the most meaningful impact.

*… moved from community nursing with the NHS [National Health Service] to an independent sector [care home] …I needed to address the issues for the whole person and not just the task, not just the clinical element of their nursing needs (P1)*

### Independence and autonomy

Discussing their roles and work, the participants indicated clearly that they were afforded more autonomy, responsibility, and independence than in NHS settings. Many ascribed this to the unique set up of social care nursing providers, and the minimisation of bureaucracy. This, they found was facilitative of action, innovative thinking, change, and meeting the needs of those they cared for.

*I find that social care nursing actually has a lot more freedom…*

*We don't subscribe to like big, lengthy terms of protocols and procedures before we can actually start doing something. We can make changes a lot quicker, and we can we're much more adaptive (P3)*

The professional responsibility for decision making needed to work in the sector was often a surprise at first, both to the nurses, and to students they support. And how this responsibility was driven by the absence of an ‘inhouse’ multidisciplinary team.

*… I think nurses in a care home or residential nursing…, have more autonomy and more responsibility because you haven't got that MDT [multidisciplinary team] team around you (P16)*

### Person centric, social ethos

The ethos of care was important to the participants. They found that nursing in adult social care enabled them to promote and protect the people they cared for to live well in society. Rather than a transactional relationship focused on a disease or disability in isolation, the nurses were able to support people to live well within their social world. They saw themselves as offering more than clinical or health-based nursing but offering a way to live and meet their needs within society.

*So, for me., ‘social’ is the biggest part of the word of ‘social care’ because it's about how that person fits into their society… It's how that how they integrate and revolve around the society that they're in and how and how we promote that and protect that (P2)*

The participants used a variety of terms to describe their approach to care including person centred, person led, relationship based, and taking a family approach. All these terms indicated that they took proactive steps to maximise peoples’ independence, giving them agency, control, and helping them co-produce their health and care provision.

*We're about people living their best life; however they want, and whatever that looks like for them… we don't focus on the medical condition, we focus very much on the person and how we can support and enable that person to live their best life: And that is a skill set in itself (P6)*

### Nursing skills

The participants described using the full range of nursing skills and attributes, incorporating these into a social model of nursing care, to meet the complexity and variety of health and wellbeing needs, often within one locality. The care described was multifaceted; they had to be dynamic and responsive to the changing needs of the people they cared for, and the services they worked within. Broadly, the skills of the nurses were identified as professional nursing skills, and business skills

### Professional nursing skills

Participants highlighted skills such as recognising and managing acute deterioration, conducting assessments, and providing positive behavioral support to prevent and manage violence and aggression. They also emphasised expertise in long-term condition management, nutritional support, wound care, end-of-life care, rehabilitation, and nursing procedures (e.g. injections and tracheostomy care). Additionally, their role encompassed health promotion and prevention, particularly in smoking cessation, addiction management, and sexual health support.

*We have to train people how to…deal with trachys [tracheostomies] and… really complex clinical skills… (P14)*

*…we've got a chap now who we are supporting to stop drinking. We've got a chap who we are supporting to stop smoking (P12)*

Nursing skills were discussed in a variety of levels including essential, specialist and advanced practice roles, and also included provision of education and training.

*I introduced… non-medical prescribing into one of the homes (P9)*

The skills of the nurses to span professional and organisational boundaries was apparent. Participants described having to navigate complex systems and partnerships, and a blurring between perceived health and social care needs.

Participant 14 articulated the complexity when discussing complex care at home (complex home care is nursing provision for people who need complex care support including people with spinal or brain injury, ventilation requirements or deteriorating conditions)

*… we've got a foot in both camps, and it doesn't always fit nicely in into a box… it's still considered social care because it's not acute care; …but it's not community care as in district nursing. I don't know quite where it fits, but I always put it in the social care box …. And the reason I put it in there is because we are dealing with everybody's daily life… It's not about making someone better…although we are putting the preventative stuff in (P14)*

### Business skills

In addition to the professional nursing skills, the participants reported requiring business and organisational management skills. Whilst many reported shift coordination responsibilities such as managing staff, others were managers, deputies or responsible for the total running of care settings when they were at work. They also were engaged with or were responsible for governance, regulatory standards, policy development, finance, and local and national organisational working.

*… the requirement to use your resources effectively, because generally it's a charity or a business-…the ability to budget appropriately to use your resources effectively (P1)*

## Consequences

The participants highlighted consequences of adult social care nursing to be related to people in services and their families. For the participants, adult social care nursing offered an avenue of living *with* a condition, whilst being empowered to be an active partner in care and life. Social care nurses spoke about recognising people as individuals, and how they integrate into their social world despite illness, injury or disability. It was felt that the contributions of social care nurses thus enable individuals to maintain a balanced and engaged lifestyle that accommodates their health needs. This support, in turn, allows people to remain in their own homes while participating in meaningful activities and social engagement.

*… it felt to some extent like there were people constrained in the… in the spaces that they were in. And what I saw were the benefits of adult social care, [people living together in one building]*... *was the ability to choose what their life could be like… (P1)*

Despite their positive experiences, the participants highlighted that a consequence of being a nurse in adult social care, was exposure to interprofessional incivility and role misinterpretation. They felt that (in some cases) their nursing registration and skills were not recognised and valued in the same esteem as nurses working in the National Health Service, conveying a sense of being ‘othered’.

*we're very often, in my opinion, seen as a less equal partner … ‘you're not a real nurse because you don't work in the hospital’ (P18)*

Whilst the nurses acknowledge the complexity around how they were perceived in wider health circles; they retained pride and were champions for raising the profile of nursing in social care.

*I just wish people could see the scale and the knowledge and the wealth of experience that we've got and celebrate it and appreciate it (P18)*

### Integrated result

Consistent with the concept analysis method, the goal was to offer a comprehensive ‘state of the art’ account at this point of time, rather than providing a definitive definition (Rodgers, 2020). This approach contributes to the evolving understanding of adult social care nursing while acknowledging the dynamic nature of health and social care provision. This study did not aim to define the attributes of social care nursing (as an activity) in isolation from the attributes of being a nurse. Instead, it recognises the link between practitioner and the practice. Drawing the theoretical literature and fieldwork voice we present a professional and public facing definition of adult social care nursing (Box 1).

**Box 1:** Professional and public definitions for Adult Social Care Nursing.

**Professional definition of adult social care nursing** Adult social care nursing seeks to support quality of life and delivers high quality care. It is built upon a philosophy of personalised care, with the recipient (and their family/carers) being active partners in co-creating how their health, independence, and wellbeing is supported, and how they can live well in their social world. Adult social care nursing is multifaceted and situates the registered nurse and the person receiving care as partners. People using adult social care nursing services are of all ages, and have a variety of needs including physical, mental health, learning disability or illness. These needs are complex and multi-faceted requiring a registered nurse to provide or oversee their care. Adult social care nursing is dynamic, independent and autonomous, relying on critical analytical and evidence-based nursing skills, in tandem with managerial and business skill development. Locations of social care nursing provision are wide ranging. This may include (but are not limited to) care homes, private homes, shared housing, non-National Health Service intermediate care settings, temporary accommodation, and services such as day centres. Adult social care nurses may employ a range of fundamental, specialist, and advanced, nursing skills depending on the people they care for. This may include (but is not limited to) long- and short-term condition management, behavioural support and communication skills, end of life care, medicines management, history taking and assessment. It encompasses direct care provision such as nutritional and hydration support, and condition specific care such as ventilatory support including tracheostomy care. Social care nurses may engage in health promotion, prevention and public health activities such as supporting health education and sexual health, smoking cessation, and addiction support. **Public definition** Adult social care nursing is the provision of comprehensive and sometimes complex care by registered nurses, to people requiring care across the lifespan. People using adult social care nursing services have a variety of needs including physical or mental health needs, having a learning disability, or an illness. This care is provided in many different settings outside of hospitals, including care homes, private homes, and day centres. Nursing in adult social care is dynamic, independent and autonomous. It relies on analytical and evidence-based nursing skills, in tandem with managerial and business development skills. The underlying ethos of adult social care nursing is centred on the person receiving the care so that it meets their specific needs; it is delivered in partnership with the person receiving the care, their families and carers; and it supports their ability to live well with their individual need, illness or disability.

## Discussion

To the best of the authors’ knowledge, this study is the first to define the concept of adult social care nursing using a concept analysis approach. It marks a turning point in how nurses working in these settings articulate their role, and helps clarify the skills, ethos and practice they undertake. Traditionally, concept analysis has relied on text-based methods to identify core features (Walker and Avant, 2011). In this study, a hybrid design was employed, combining analysis of contemporary literature with insights from nursing professionals working in social care settings. This approach reflects the understanding that concepts are both empirical and abstract - grounded in observable experiences while simultaneously shaped through subjective interpretation (Chinn and Kramer, 1991).

Despite being registered health professionals, adult social care nurses work within a context where distinctions between ‘health’ and ‘social care’ needs are blurred (Simpson et al., 2023). In England, this ambiguity is reinforced by the absence of clear legal definitions specifying what constitutes a health need as distinct from a social care need when needs are assessed (Department of Health and Social Care, 2022b). In practice, attempts to categorise needs often rely on frameworks that separate the ‘clinical’ or ‘biological’ from ‘non-biological’. Such binary distinctions risk oversimplifying the complex, interdependent nature of needs encountered in adult social care. Adult social care nurses adopt holistic, person-centred approaches, recognising that health needs may directly shape social care needs, and that unmet social care needs can in turn exacerbate health conditions.

Adult social care nurses frequently experience role confusion, and their nursing skills are often wrongly perceived as “second rate” or a “lower option” compared to colleagues working in the National Health Service, which has become synonymous with healthcare provision (Thompson et al., 2018). This research highlights that adult social care nurses are highly skilled, autonomous professionals who uphold the standards of practice set by their regulatory body. Our work builds on existing research by Cornes & Manthorpe (2022), and Steils et al. (2024), highlighting that nurses in social care manage a significant level of complexity, navigating multiple organisational, and structural processes to deliver evidence-based nursing care. Many develop business acumen beyond traditional leadership and management principles more aligned with senior/chief nurses in the NHS

Participants in this study expressed mixed views on the usefulness of defining their roles based on the setting in which they work. There was no clear consensus on whether the label 'adult social care nursing' served as a help or a hindrance. This echoes the complexity found by Steils et al. (2024) in terms of how nurses in social care hold varying in positions around being termed a “nurse” first and foremost, “social care nurse” as a speciality or distinct field of practice. It is well recognised that naming convention for nursing roles is subject to lack of standardisation, with a plethora of titles and role descriptors being used across services and employers (Harrison, 2018), despite typical naming conventions being formed of professional registration, scope of practice/speciality, seniority and skill set (Grundy-Bowers, 2018). Research to date has focussed on alignment of role titles across National Health Service employment and specialist levels of practice (Grundy-Bowers, 2018; Leary et al., 2017), with an absence of social care settings considered. And whilst this research did not aim to understand the variation or array of nursing titles, it echoes the premise that nurses in social care may feel their role and identity is not well understood or consistently named.

Our analysis adds to the discourse around professional identities and how these impact on the ecosystem of health and social care. The participants’ experiences reflect broader systemic attitudes that undermine the value of adult social care nursing and present barriers to interprofessional respect and collaboration. This highlights the complexity of how social care nurses' professional identities are shaped not only by their immediate roles but also by the layered and often hierarchical systems in which they operate. Any discussion aimed at supporting, retaining, or developing this workforce must consider these multifaceted influences on role identity, recognition, and professional legitimacy. Factors that influence the occupational status of nurses in social care include characteristics of their roles which are subjected to judgement by society (Thompson et al., 2018), are linked to perceptions of authority and knowledge (Zhou, 2005), and mediated by recognition and status applied by the dominant sociocultural entity (Bourdieu, 2002). For social care nurses, this mediation happens at the intersection of political, service provision, and educational determinants. Recognising this triad of influence, there is a need for powerful leaders to signal the knowledge base and authority of nurses working in social care as on equal footing with National Health Service employed nurses- as their shared professional registration permits. This concept analysis supports this assertion by providing a definition that attends to both the knowledge base and legitimate professional authority afforded to them as registered nurses.

Globally, social care nursing typically falls within long-term care service models. The European Commission defines Long-Term Care Services as "a range of services and assistance for people who, due to mental and/or physical frailty and/or disability over an extended period of time, depend on help with daily living activities and/or require permanent nursing care" (European Commission, 2021, p.426). In response to growing demands for nursing care, countries such as Japan, Germany, and South Korea have introduced mandatory long-term care insurance (Costa-Font and Rault, 2022), while the Netherlands employs a dual insurance model combining mandatory long-term care insurance and health insurance. This system covers nursing care costs depending on whether care is provided at home or in a nursing home. In contrast, countries like Scotland, France, Australia, and Spain have tax-funded systems, while England adopts a means-tested approach.

These international variations underscore the complexity of defining and standardising the role of social care nurses, particularly in contexts where social support, personal care, nursing care, and institutional care are separated. Our analysis suggests that, even within a single system like England, these structural distinctions can influence professional identity, role recognition, and interprofessional collaboration. Placing adult social care nursing within this global perspective highlights the challenges of coordination, accountability, and valuation of nursing work across diverse care models (Costa-Font and Rault, 2022).

This fragmentation inevitably affects nurses working within health and social care. In the England, this is particularly evident in the structural divide whereby social support is the responsibility of local authorities, while healthcare provision falls under the remit of the National Health Service (Thompson et al., 2018). While this separation of services highlights the nursing role within social care provision, it fails to address the unique structure of health and care services, and the varying funding models applied across the life course. Long-term care has traditionally been a focal point for nursing in social care settings, especially with an ageing population necessitating increased care services. However, it is important to acknowledge that nursing in social care does not solely address the needs of older individuals requiring long-term care. It also involves short-term reablement or rehabilitation (Cornes and White et al., 2018), where nurses play a critical role in promoting independence, reducing dependency, and restoring health. This recognition reinforces the importance of clarifying the concept within specific health and social care contexts.

There is now an overt drive to support nurses in social care from a political, educational, and service perspective within the UK- signalled by the appointment of the first Chief nurse for social care in England, and professional recognition of social care as an area of specialist practice by the Nursing and Midwifery Council and Queens Nursing Institute (Nursing and Midwifery Council, 2022; Queens Nursing Institute, 2022), alongside education institutions starting to understand mechanisms to support increased sociocultural acceptance of social care nursing.

Pre-registration education of nurses is largely driven by National Health Service workforce calculations and perspectives. Watson et al. (2020) highlighted that in respect to care home nursing, the absence of placements in social care settings can send implicit negative signals that this is not an equally important area of nursing practice. This, in turn, may lead students to draw conclusions about what this work involved without any practical or theoretical basis, instead relying on dramatized media portrayals, family experiences, and the attitudes of others. Watson et al. (2020) therefore recommend inclusion in the curriculum to counter negative perceptions built on absence of information, and a better representation of the positive aspects of nursing in care homes.

The Nursing and Midwifery Council broadly support expansion to social care placements (Nursing and Midwifery Council, 2025), but a clear mandate to education providers to include social care placements and learning activity remains lacking. Despite this, innovative curriculum design and shift to include social care is growing, with first destination pathways focussing on community and social care nursing, and care home internship models of education emerging (Tiplady et al., 2018). More recently, there have been visible, tangible shifts towards better inclusive education, recommended by Stelis and embodied in the Skills for Care (2025) placement strategy supported by the Council of Deans of Health. This growing emphasis on social care nursing within education helps strengthen its professional legitimacy and authority and is likely to influence perceptions of the field. However, further research is needed to understand how these changes impact higher education institutions, curricula, and the attitudes of students and registrants towards social care nursing.

This study provides the first conceptually grounded definition of adult social care nursing, integrating literature with the lived experiences of nurses in these settings. It makes visible a field of practice that has historically been marginalised, clarifies its knowledge base and scope, and underscores the autonomy and complexity of the work undertaken. Recognising adult social care nursing as a distinct and legitimate field is essential for workforce sustainability, interprofessional collaboration, and the delivery of person-centred care. This analysis establishes a necessary foundation for supporting and valuing this critical workforce.

### Limitations, and recommendations for future research and practice

Within the existing literature, professional work profiles were often poorly reported, with many studies focusing on registered managers of services without detailing their specific professional registration or roles (i.e. manager *and* nurse registration). The complexity of identifying registered nurses limited phase one. Regarding fieldwork, the participant sample consisted of mainly senior professionals, which influenced the diversity of frontline perspectives. Despite this, the study was able to capture a wide range of role profiles, providing valuable insight into various experiences.

This study has highlighted a critical dearth of understanding and articulation in policy and literature around what is meant by social care nursing. There is a clear need to attend to a broader narrative of Social Care Nurse provision past care home locations, including it in the wider concepts of community and neighbourhood health services, and across the life span. While this study captured data on some of the complex factors shaping understandings of social care nursing, unpacking this in depth was beyond its scope. Future studies should explore this in more depth. In addition, there was limited discussion of wider potential locations of social care nursing such as inclusion health services and prisons. It is recommended that researchers consider these areas in future research to illuminate the scope and impact these nurses have on wider societal health and social wellbeing.

Understanding and recognition of adult social care nursing as a distinct specialist area of practice needs to be embedded in pre-registration nurse education. Our study indicates that, while there is a welcome move in this direction in practice, further curriculum review is required to ensure that media, imagery, placement provision, and theoretical content achieve a more balanced representation of National Health Service nursing and social care nursing. We therefore recommend further research to examine the impact of these changes on the knowledge, professional perceptions and employment decisions of nurses.

## Conclusion

This paper offers the first formal definition of nursing within adult social care, which is intended to serve as a springboard for further discussion and development. This definition holds global relevance, as it highlights the diverse skills, knowledge, and attributes that nurses bring to social care. It provides a flexible framework that can be adapted across international social care systems, promoting recognition of nursing roles beyond the context of large-scale statutory healthcare provision. Ultimately, by defining the concept of adult social care nursing as a professional practice, this paper illuminates the complexity and multifaceted nature of the role.

While this definition may evolve in line with nursing practice and health and social care agendas, it provides a valuable and necessary starting point. This only raises the profile of nurses within the social care sector but also highlights the broad skill set they bring to their practice., The definition emphasises the integral role of nurses in social care in delivering integrated and high-quality care. This serves to clarify the complexity of the nursing within social care, positioning nurses as essential contributors to person-centred care. Their role and contributions should be acknowledged within the broader context of health and social care reform.

## Funding

This work was supported by RCN Foundation. Grant number 1625,025

Tri-morbidity is the presence of mental and physical ill health combined with substance abuse (Bradley, 2018)

## CRediT authorship contribution statement

**Claire Pryor:** Writing – review & editing, Writing – original draft, Supervision, Resources, Project administration, Methodology, Investigation, Formal analysis, Data curation, Conceptualization. **Siobhán Kelly:** Writing – review & editing, Writing – original draft, Project administration, Methodology, Investigation, Formal analysis, Data curation, Conceptualization. **Vanessa Heaslip:** Writing – review & editing, Writing – original draft, Project administration, Methodology, Investigation, Formal analysis, Data curation, Conceptualization. **Deepa Korea:** Writing – review & editing, Conceptualization. **Melanie Stephens:** Writing – review & editing, Writing – original draft, Conceptualization.

## Declaration of competing interest

The authors declare that they have no known competing financial interests or personal relationships that could have appeared to influence the work reported in this paper.

## Appendix Supplementary materials

Image, application 1

## Footnotes

1 Tri-morbidity is the presence of mental and physical ill health combined with substance abuse (Bradley, 2018)
