# Supplementary material for: Adult social care nursing: A hybrid concept analysis
Source: Int J Nurs Stud Adv. 2026 Jan 24;10:100491. doi: 10.1016/j.ijnsa.2026.100491 (PMC12908038; doi:10.1016/j.ijnsa.2026.100491)
Supplement: Supplementary file 1 [file mmc1.docx]

Supplementary file 1 Example of data extraction

 
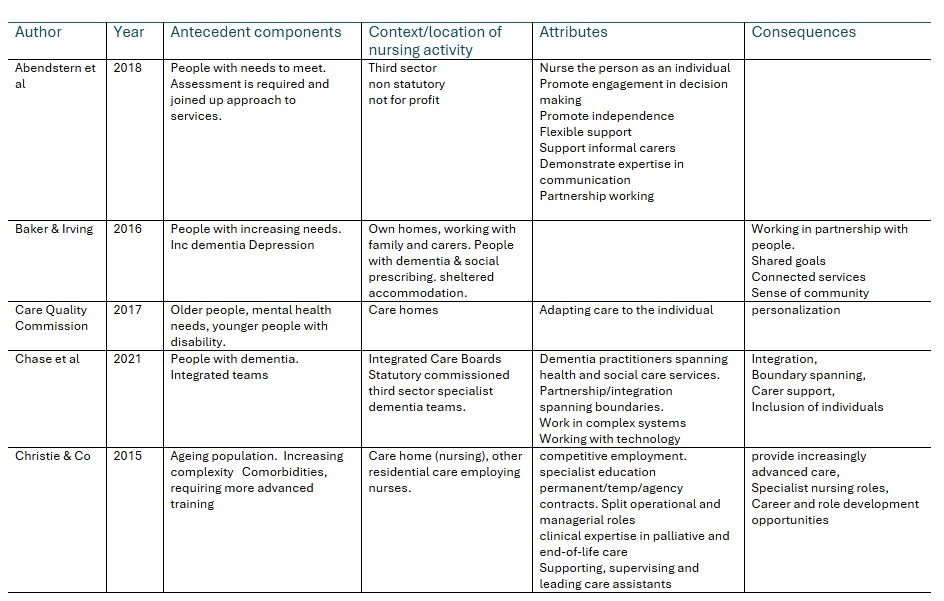


Supplementary file 2:  An example of thematic antecedent formation

| **Participant** | **Quote** | **Context** | **Meaning** - **initial coding** | **Refinement** |
| --- | --- | --- | --- | --- |
| P1 | Any adult who is not in an NHS [National Health Service] residential type setting. So that could be learning disabilities. It could be mental health, it could be People with acquired brain injuries, it could be anybody. It's absolutely anybody.  So, I managed a mental health hospital, detained patients, tribunals, all that type of stuff.  But a large element of it was still social care, because we were still sitting in the social care sector, even though we were a hospital, ... And then there was slow stream rehab on the other side, who were getting them ready to go into the community. | Discussing who they think uses Social Care nursing      Discussing their perception of Adult Social Care and settings. Because ethos was social rehabilitation + health need, considers it Adult Social Care Nursing | Not National Health Service residential  Adult Social Care provides care to anyone who needs nursing care.  Including in settings traditionally seen as health? | Antecedent:  Care needed for a range of peoples’ needs such as Learning disability/Mental Health /Acquired brain injury.  Care needed in a variety of settings including some non-traditional social care settings such as independent hospitals if the focus is on social rehabilitation with the inclusion of a need that required qualified nursing care and support |
| P4 | For those that you care for, and I'm not just talking about elderly care because I have services that have mental health, We have a, dementia communities. We have young persons, disabilities. So, I'm looking at it from the perspective of a wide range of nursing services that I support personally | Discussing their perception of what Adult Social Care Nursing covers | Care provision is people who are elderly, have mental health needs, require dementia support, young people and people with other disabilities. | Adult Social Care Nursing provides care for all adults across their lives who require nursing care- including younger adults, |
| P3 | But yeah, I always think about the likes of domiciliary care, about adult care homes, care homes for people with learning disabilities, younger adults. | Discussing their perception of what Adult Social Care Nursing covers | Expanding from care at home, into care homes for all adults | Care in home and care homes for all adults across their lives including younger adults, and people with Learning Disability |
| Theme: Potential Antecedent Statement:  Care is provided for a range of individuals, across the life course- in a range of settings (Younger people, adult, older, Mental health needs, Learning disabilities) P10; P11; P12; P13; P14; P17; P18; P19 | | | | |
